# The Use of Tethered Bilayer Lipid Membranes to Identify the Mechanisms of Antimicrobial Peptide Interactions with Lipid Bilayers

**DOI:** 10.3390/antibiotics8010012

**Published:** 2019-01-30

**Authors:** Amani Alghalayini, Alvaro Garcia, Thomas Berry, Charles G. Cranfield

**Affiliations:** School of Life Science, University of Technology Sydney, Ultimo, NSW 2007, Australia; Amani.Alghalayini@student.uts.edu.au (A.A.); alvaro.garcia@uts.edu.au (A.G.); Thomas.Berry@student.uts.edu.au (T.B.)

**Keywords:** antimicrobial peptides, tethered bilayer lipid membranes, electrical impedance spectroscopy

## Abstract

This review identifies the ways in which tethered bilayer lipid membranes (tBLMs) can be used for the identification of the actions of antimicrobials against lipid bilayers. Much of the new research in this area has originated, or included researchers from, the southern hemisphere, Australia and New Zealand in particular. More and more, tBLMs are replacing liposome release assays, black lipid membranes and patch-clamp electrophysiological techniques because they use fewer reagents, are able to obtain results far more quickly and can provide a uniformity of responses with fewer artefacts. In this work, we describe how tBLM technology can and has been used to identify the actions of numerous antimicrobial agents.

## 1. Introduction

Antimicrobial peptides (AMPs) are of increasing interest as potential lead candidates for treating infection because they are thought to be more impervious to antibacterial resistance mechanisms [1]. One of the most rapid and increasingly effective methods for testing the actions of antimicrobials that target microbial membranes is to use tethered bilayer lipid membranes (tBLMs) in association with electrical impedance spectroscopy (EIS). In this review, we outline the advantages of this technique compared to other lipid membrane techniques and describe how tBLMs, in conjunction with EIS, have been used to rapidly and reliably identify the actions of many AMPs.

The desire to study membranes and determine changes in their physical and electrical properties encouraged the development of artificial lipid bilayer systems. The first of these was developed by Mueller et al. [2] and was subsequently named black lipid membranes. These model membranes consisted of a painted phospholipid bilayer across a small aperture, usually 1 mm in diameter, between two chambers that held aqueous solutions. This system provided a planar lipid bilayer that could be probed with electrical or basic optical techniques. The name black lipid membrane was derived from the optical technique used to determine when the bilayer had formed. Once the film had been painted on, the mixture would begin to thin and become a single spanning bilayer, this would then cause interference of reflected light and render the membrane opaque [3]. The fact that no underlying support existed meant that the membrane-associated proteins were able to function without interference. However, non-polar solvents are required in the manufacture of these membranes which alter the integral properties of the lipid bilayer [4].

Attempts to find alternatives, which abolished the solvent artefact in the membrane, led to the production of phospholipid bilayers supported by solid substrates. Tamm and McConnell (1985) were the first to describe this technique, which enabled them to create extremely stable bilayers supported by a number of different substrates (silicon oxide wafers, glass coverslips and quartz slides). The ability to adhere to these surfaces was believed to originate from the silicious materials, which have deprotonated free silanols at the surface at neutral pH [5]. Even though they had been using zwitterionic phospholipids, an apparent interfacial potential was established allowing the surface of the bilayer to be attracted to the surface of the substrate. However, as acknowledged by Tamm and McConnell themselves, the fact that the substrate was adjacent to the bilayer meant that a very thin water layer existed between the phospholipid and the solid support [5]. This meant that the incorporation of transmembrane proteins was hampered by the high probability of the protein interacting with the substrate surface [6,7]. This could cause both immobilization of the protein with inhibition of function or cause denaturation of the protein in the vicinity of the contact point between protein and substrate [8]. This has particularly been noted in the inability of integral proteins to diffuse freely through solid-supported bilayer systems [9,10,11,12]. Not only is the inclusion of integral membrane proteins difficult in solid-supported bilayer systems, but the study of the electrical properties of these membranes is difficult to perform when the aqueous reservoir between the membrane and substrate is so small [13]. Thus, a supported lipid bilayer with an appropriate reservoir space to allow accommodation of transmembrane proteins and a free diffusion of ions was pursued.

Attempts at constructing novel solid-supported membranes that contained a larger reservoir were made by laying phospholipid bilayers onto water swellable polymer cushions sitting between the solid substrate and bilayer [7,14,15,16]. The silicious materials used in these studies enabled the construction of polymer cushions that adhered directly to either the surface itself or a functionalized surface, through silinisation, using either acrylamide, dextran or agarose substrates. Silinisation could be used on metal solid substrates which could be incorporated into impedance spectroscopy systems to determine the electrical properties of the membrane [17]. Further modification of the polymer cushion substrates themselves allowed adherence to substrates, such as gold, through sulphur coordination [18].

**Figure 1:**
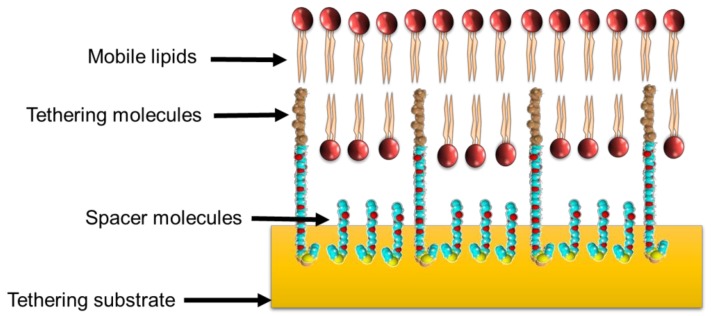
A basic tethered bilayer lipid membrane architecture. The use of membrane-tethering molecules and spacer molecules creates a reservoir between the membrane and the substrate to provide space for the transport of ions and the insertion of extended membrane-bound peptides or proteins.

It was noted that applying a phospholipid bilayer onto a polymer cushion did not mitigate inherent problems associated with membrane stability [19]. The issue of stability was circumvented with a new functionalized polymer cushion that created anchor points for customized lipids to attach and, in turn, tether the membrane to the cushion through hydrophobic forces [19]. This improvement in stability did not abrogate the continuing issue of a reservoir that restricted ion diffusion between the solid substrate and the phospholipid bilayer. A new membrane technology from Australia was reported by Cornell et al., (1997), consisting of a membrane separated from the surface of the substrate through the use of double-length reservoir half-membrane spanning diphytanyl (DLP) ethylene glycol tethers. This enabled the formation of a lipid bilayer around the end of the DLP through the half-membrane spanning component of the DLP chain [20]. The other end of the DLP was firmly attached to a gold substrate surface through sulphur–gold coordination chemistry. This system took the concept of tethering or anchoring the membrane to the solid substrate surface described by Beyer et al., (1996) and removed the polymer cushion, thus creating a much larger reservoir space for transmembrane protein insertion and free ion diffusion (Figure 1). This optimized reservoir space was enhanced by mixing in polar spacer molecules, which prevented the formation of a monolayer beneath the membrane by providing lateral separation of the tethers [13]. This system provides an enormous amount of flexibility in the composition of the phospholipid bilayer, as any mixture of phospholipids able to create a bilayer could be studied. Since then, several studies have investigated and described alternative tethering components [21,22,23,24,25,26,27,28,29,30] and substrates such as liquid mercury [31,32] to create stable supported bilayers. Southern hemisphere researchers of note in the development of new tethered bilayer architectures are researchers from the McGuillivray group at the University of Aukland, and the Köper group at Flinders University, in South Australia.

The variety of novel approaches to the design of tethering systems to maintain stable and dynamic bilayer systems provides a large toolkit to create explicit solutions for particular biological problems such as identifying how antimicrobials interact with lipid bilayers. This review does not seek to identify all the research into AMPs using tBLMs, rather, it seeks to detail what tBLM technology can do to identify the actions of AMPs.

## 2. Models of AMP–Lipid Membrane Interactions

In studying the interactions between lipid bilayers and antimicrobial peptides, a variety of models have been proposed to identify the exact mechanism of action associated with these interactions. Each family of peptides appears to interact with lipid bilayers in a unique manner and most current interactions have been identified as either pore forming, intrinsic pore modulating or having surfactant-like properties that induce membrane rupture or lysis [33,34,35,36,37,38,39,40,41,42,43]. Individual types of peptides may not conform to a particular mode of action or have the qualities of each model, with some peptides not fitting within any of these models [35,44,45,46]. The proposed mechanisms of action of AMPs are briefly summarized here, along with a subsequent explanation of how tBLMs can be used in conjunction with swept frequency electrical impedance spectroscopy (EIS) to distinguish between these mechanisms.

### 2.1. Barrel-Stave Model

**Figure 2:**
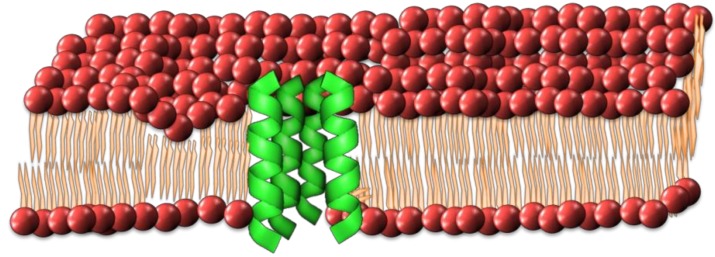
Schematic of how individual peptides might form the barrel-stave pore configuration in a cell membrane.

This model describes the formation of an ionic conductive pore through the membrane resembling a barrel with individual peptides forming the staves. [33,47,48]. The process of pore formation involves the insertion of peptides through the lipid bilayer, along with a successive aggregation to form a transmembrane channel (Figure 2). An amphipathic peptide structure is required, in which the hydrophobic regions are aligned with the hydrophobic inner domain of the lipid bilayer and the hydrophilic regions face the pore lumen. This creates a hydrophilic channel through the lipid bilayer allowing the free passage of ions and solutes through the membrane [47,48]. The diameter of the pore lumen is intrinsically linked to the number peptides recruited, with larger groupings of peptides forming larger pores, resulting in increased leakage of cell contents and potentially leading to cell death [35].

### 2.2. Interdigitated Peptide Toroidal Pore Model

**Figure 3:**
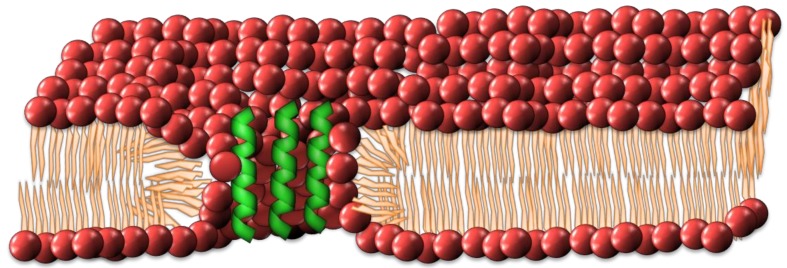
The toroidal pore model has the antimicrobial peptides (AMPs) interdigitated between lipid head groups as part of a pore within the membrane.

The barrel-stave model has evolved into different forms, with a number of antimicrobial peptides not conforming to the rudimentary barrel-stave model, namely magainins, protegrins and melittin [48,49,50,51,52]. An alternate method was put forth by Matsuzaki et al. [49]. The toroidal model differs from the barrel-stave model in that the lipid headgroups of the bilayer participate in the formation of the pore. The structure of this pore demands that the inner and outer leaflets bend in such a way as to form a pore composed of interdigitated peptide and phospholipid headgroups (See Figure 3). In contrast to the barrel-stave model, the peptides remain associated with the headgroups of the lipids and do not permeate through the hydrophobic chain regions [33]. 

### 2.3. Carpet Model

**Figure 4:**
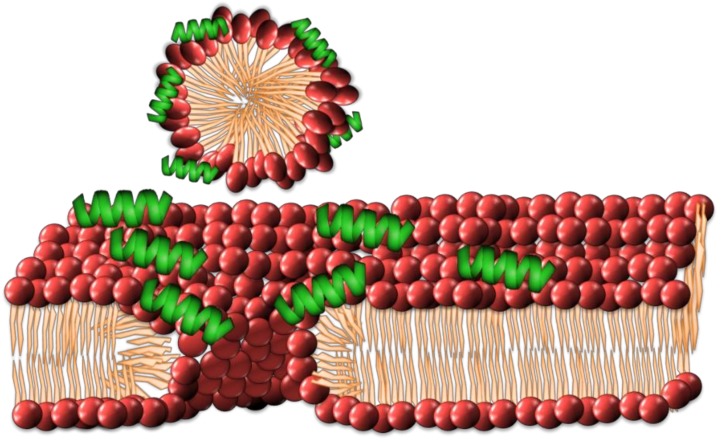
The carpet model of cell membrane disruption has the accumulation of amphipathic AMPs as a ‘carpet’ across the membrane, eventually promoting to the micellization of individual lipids creating membrane defects.

This model describes the gathering of peptides at the lipid–water interface of the lipid bilayer, attracted there through electrostatic forces. It was first theorized to describe the interactions of the peptide dermaseptin [53]. This model has also been used to describe the interactions of peptides such as ovispirin and melittin [33,54,55]. As the concentration of peptides increases at the lipid–water interface, they form a ‘carpet’ across the surface of the bilayer (Figure 4). The peptides are reported to permeabilize the membrane by disrupting phospholipid packing and are suggested to have surfactant-like qualities in high concentrations, leading to the removal of small segments of the bilayer through micellization [40].

### 2.4. Intrinsic Pore Modulation Model by Changing the Critical Packing Paremeter (CPP)

A more recent model of peptide–lipid interactions, suggested by Australian researchers amongst others, describes the process in terms of altering the critical packing parameter (CPP) of the bilayer and, thereby, altering the size of membrane pores already present in the membrane [36,37,56,57,58,59,60].

**Figure 5:**
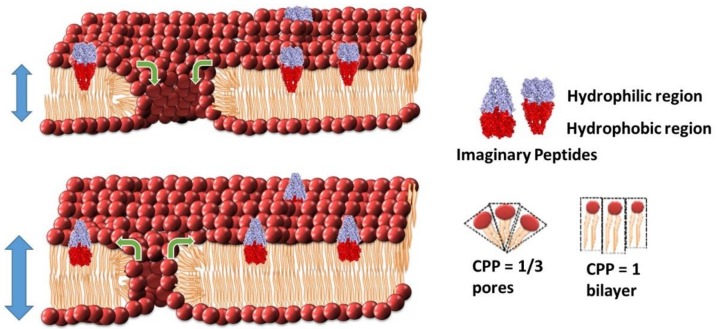
The Critical Packing Parameter (CPP) model of toroidal pore modulation by antimicrobial agents. This model predicts that the shape of peptides influences the lipid packing arrangement leading to either an increase in intrinsic membrane pore radius and an overall slight thinning of the membrane, as more lipids diffuse into pore regions, or a decrease in the intrinsic membrane pore radius with a thickening of the membrane as more lipids diffuse out of the pore regions.

The CPP concept was first introduced by Jacob Israelachvili during his time at Australian National University [61,62]. This model aims to predict the morphology of lipids based on the ratio of the lipid head groups’ surface area (*a_0_*) and the hydrophobic lipid chain lengths (*l*) with the overall volume of the individual lipids (*v*), such that CPP = *v/a_0_l*. Within a planar bilayer, the overall CPP = 1. An overall CCP = 1/3 describes a micelle but also describes the CPP of the lipids that make up the curved regions of a toroidal pore (Figure 5) [36]. The CPP-pore modulation model suggests the interaction of peptides is influencing the size of pre-existing pores or defects found within the lipid bilayer [36]. Upon interacting with the lipid bilayer, the peptides disrupt the packing of the surrounding lipids causing changes in the overall CPP of the bilayer. This interaction of lipids and peptides can cause a change in the effective head group surface area (*a_0_*) compared to the lipid chain length (*l*), either increasing or decreasing the CPP depending on the geometry of the peptide in the bilayer. When the CPP of an individual lipid drops below unity within a lipid bilayer, it lacks sufficient laxity for lateral movement beyond the existing boundaries. This model suggests that the need for space may be resolved through movement of lipids into or out of existing toroidal pores where the CPP = 1/3, and/or with an alteration in chain length to compensate for the change in the surface area in the planar bilayer sections where the CPP has to remain equal to one.

### 2.5. Identifying Mechanisms of Membrane Interaction Using EIS Techniques

Swept frequency electrical impedance spectroscopy is the method most commonly used to identify the ionic conduction across a tethered bilayer lipid membrane. It also characterizes the membrane capacitance, which identifies changes in membrane thickness and/or water content. These changes in membrane thickness and/or water content are derived from the geometric properties of a capacitor. In its simplest form, a lipid bilayer can be modelled as a parallel-plate capacitor, itself in parallel with a resistor. The membrane thickness pertains to the distance between the two plates of the capacitor and the water content determines the relative permittivity (*ε_r_*). Thus, as membrane capacitance increases, the membrane thickness (distance between the plates) decreases and/or the water content (relative permittivity between the plates) increases. The opposite is true when the membrane capacitance decreases. When studying the interactions of antimicrobial peptides with lipid bilayers, the ability to identify changes in either or both of these values can assist in isolating the mechanisms of antimicrobial interactions.

**Figure 6:**
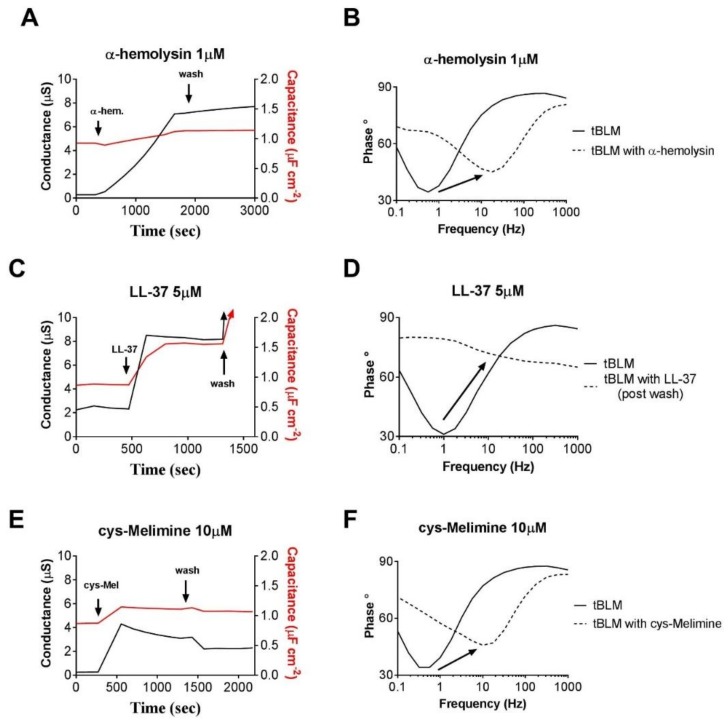
The tethered bilayer lipid membrane (tBLM) responses presented here are actual data obtained in the laboratory of the authors. (**A**) Membrane conduction and capacitance responses to the membrane ion channel forming antibiotic α-hemolysin. (**B**) Phase versus frequency (Bode Plot) before and after addition of the antibiotic α-hemolysin. The phase minima typically shift to higher frequencies with little increase in the phase angle. (**C**) Membrane conduction and capacitance responses to the human defensin peptide, LL-37, that causes membrane lysis. The responses are particularly evident after the membrane undergoes a wash step which induces mild sheer stress at the membrane. (**D**) Phase versus frequency (Bode Plot) response of LL-37. The phase minima typically shift to higher frequencies with very large increase in the phase angle. This phase signature is evidence of the tBLM undergoing irrevocable disruption. (**E**) Membrane conduction and capacitance responses to the AMP cys-Melimine [37]. A mild increase in membrane conduction is evident at relatively high concentrations of the AMP with a small change in membrane capacitance. The responses are partially reversed after washing. Note that the concentration of the AMP is 10 times larger than that for the ion channel α-hemolysin (Figure 6A). (**F**) Phase versus frequency (Bode Plot) response of the AMP cys-Melimine. The phase minima typically shift to higher frequencies with only a small increase in the phase angle.

A large increase in membrane conduction, with limited or only a small change in membrane capacitance, indicates the formation of ion channels within the membrane, particularly if the conduction effect does not readily disappear with subsequent wash steps. This type of response is typical of pore forming AMPs where it would be expected that insertion and aggregation of the peptides in the membrane would disrupt the packing of adjacent lipids, with a reduced influence on packing structure at further distances from the annular ring [63]. A classic example of this would be channels formed by the antibiotic α-hemolysin (Figure 6A,B) [64]. We can see a significant increase in conduction across the membrane with an associated small increase in membrane capacitance (Figure 6A). The small membrane capacitance change in this case can be attributed to the localized disruption of phospholipid packing in the annular ring around the α-hemolysin as determined by neutron reflectometry [65].

A large increase in membrane conduction that is also associated with a large increase in membrane capacitance is suggestive of antimicrobials having a lytic or surfactant-like effect. It is expected that these effects would be concentration dependent and that reaching a particular concentration threshold would enable sequestration of lipids from the bilayer through micellization. These effects are typically irreversible with subsequent wash steps and often washing will compound the effects. The increase in membrane capacitance in this case is suggestive of the membrane getting thinner, probably due to the removal of lipids from the bilayer as a result of the surfactant-like activity of the AMPs. An example of this response is the human cathelicidin AMP LL-37 [66,67] (Figure 6C,D). The initial interaction of this peptide can be described by the “carpet model”, with disruption of the membrane due to intercalation of the peptide with lipid headgroups [68,69] and an associated decrease in membrane capacitance. However, as has been noted [70], peptides that follow the “carpet model” can possess a threshold concentration at which significant disruption of the phospholipid bilayer is observed. This would then manifest itself as a large increase in bilayer capacitance as the surfactant-like effect of removing lipids thins the bilayer.

In many cases, AMPs can be added that are known to be too small to traverse the membrane yet can still induce increases or decreases in overall membrane conduction. In these sorts of responses, there are typically only very small changes in membrane capacitance, if any. Typically, to some degree, these AMPs can be readily washed out of the membrane (Figure 6E,F). There have been numerous reports of AMPs or peptidomimetics that induce these responses [37,60,67,71,72]. The change in membrane conduction in these cases has been assigned to a modulation of intrinsic membrane pores via a rearrangement of the packing of the lipids according the CPP model (Figure 5).

A difficulty arises in comparing the concentration dependence of membrane disruption in tBLM systems to the minimal inhibitory concentrations derived from in vitro bacterial growth experiments [73]. Given that EIS measurements are limited to the antimicrobial peptides’ ability to modulate the physical structure of the lipid membrane, the fact that these peptides have other purported antimicrobial properties unrelated to membrane disruption must be considered [74]. Other considerations in the use of tBLMs include the ratio of tethered lipids to freely diffusing lipids and the relative volume of the reservoir region between the substrate and the bilayer. In principle, it is better to have as few tethered lipids, compared to freely diffusing lipids, as possible, and to have as large a reservoir region as possible to enable the free passage of ions [75].

## 3. Antimicrobial–Lipid Membrane Interactions Investigated Using tBLMs

### 3.1. Testing the Lipid Specificity of AMPs

There have been numerous studies that have shown how antimicrobials have a preference for negatively charged lipids over zwitterionic lipids [37,64,76]. The relative high number of positively charged amino acids reported in AMPs confer this preference through electrostatic attraction. However, there is not always a correlation between a peptide’s charge and its affinity for negatively charged lipids. The bespoke antimicrobial peptide chimera of melittin and protamine, melimine, and its analogs have been investigated by Australian researchers using tBLMs and their actions have been described according to the CPP model as mentioned above [37]. Despite the presence of multiple positively charged amino acid residues in these peptides, there was little correlation with their interactions in tBLMs comprised of a high percentage of negatively charged lipids. Instead, a correlation was made according to the number and location of hydrophobic peptide residues. 

Researchers in Sydney, Australia, studied a group of bespoke biphenyl peptidomimetics using tBLMs to identify their mechanism of action. Zwitterionic and negatively charged lipids were employed to determine how headgroup charges may modulate the electrostatic interactions of these peptidomimetics [72]. For each synthetic peptidomimic, their capacity to disrupt tBLMs was compared to their minimum inhibitory concentration (MIC) against bacteria and was found to not consistently correlate. This was used as evidence that the antimicrobial mechanisms of these peptidomimetics were not necessarily as a result of their actions on negatively charged bacterial membranes.

Other small molecular antimicrobial peptidomimics, N-naphthoyl-phenylglyoxamide-based and N-sulfonylphenylglyoxamide-based antimicrobial peptides, were investigated to determine whether the mechanism of action was related to their capacity to disrupt phospholipid membranes [60,67]. In each case, their membrane interactions were described using the CPP pore modulation model and only partially correlated with the MIC observed in bacterial experiments.

In the case of the naturally occurring Kalata B1 and Kalata B2 cyclic antimicrobial and insecticidal peptides (of which researchers from the David Craik laboratory at the University of Queensland are recognized world leaders [77]), their specificity for membranes that contain phosphotidyl ethanolamine (PE) lipids was confirmed using tBLMs [56]. This specificity was derived from a binding pocket within the peptide containing both negatively and positively charged amino acid residues which specifically targeted PE lipids and had little affinity for negatively charged lipid headgroups [78]. Electrical impedance spectroscopy identified large changes in the membrane capacitance and membrane conductance suggesting activity of these cyclotides was due to a surfactant-like mechanism rather than the previously reported pore forming mechanism [79].

These results demonstrate the capacity of tBLMs in conjunction with electrical impedance spectroscopy to elucidate the membrane disruptive properties of novel peptidomimetics and provides valuable information regarding affinities for particular lipid compositions. Further, tBLM technology used in this way permits a rapid characterization of peptide/membrane interactions and provides a basis for implementing an iterative development of synthetic peptides.

### 3.2. Voltametric Techniques to Explore Antimicrobial Interations

As well as electrical impedance spectroscopy, other electrical techniques, such as ramped or pulsed amperometry can be employed to identify the actions of various antimicrobial agents. The amphipathic antimicrobial peptide trichogin GA IV (TCG) was investigated using applied potential steps at 50 mV increments [80]. Using this technique, the researchers determined the required membrane potentials for incorporation of the peptide. The researchers also employed cyclic voltammetry and identified that TCG has a voltage-gated behavior similar to the fungal peptaibol peptide alamethicin.

The use of potential steps and ramped amperometry has also been employed to identify how peptides can make use of membrane defects to incorporate into membranes. Led by Australian researchers, Cranfield et al. (2014) showed that by rapidly increasing the potential across the tBLM, they could induce a detectable electroporation effect. They were then able to show that defects caused by electroporation induced an increase in the activity of the African clawed frog antimicrobial peptide PGLa [64].

### 3.3. Bacterial Surface tBLM Mimics

Significant effort has gone into creating tBLMs that better mimic the actual surface of bacteria. There have been efforts to incorporate commercially supplied lipids from *E. coli* sources in tBLMs with some success [37], but these membranes do not have the lipopolysaccharide (LPS) layer that is associated with bacterial membranes. Recently, however, Andersson et al. (2018) from the Köper group at Flinders University in South Australia in collaboration with researchers from the Australian Nuclear Science and Technology Organisation (ANSTO) were able to fuse liposomes of a lipopolysaccharide purified from *E. coli* onto a monolayer of tethering lipids [81]. They were then able to test their LPS–tBLM using the antimicrobial colistin sulfate and were able to elicit a change in the membrane structure as evidenced by neutron scattering and EIS measures.

Spencelayh et al., (2006) were able to form tBLMs that incorporated Lipid I and Lipid II, which are precursors to the peptidoglycan layer of bacterial cell walls. They were then able to test the glycopeptide antibiotics vancomycin and ramoplanin against these tBLM architectures. These types of antibiotics interfere with the formation of the peptidoglycan coatings that protect Gram-positive bacteria from lysis. Surface plasmon resonance and EIS were employed to measure changes in membrane thickness as a result of adding these antibiotics. Significantly, purified inner *E. coli* membranes were used to form these tBLMs [82].

Outer membrane protein F (OmpF) is one of the porin transmembrane proteins found in *E. coli* outer membranes and is a target for antibiotics such as colicin N [83]. Stora et al. (1999) were able to incorporate OmpF into tBLMs and demonstrate that colicin N was able to reduce overall membrane conduction as a result [84]. The same group were later able to self-assemble tBLMs containing cysteine mutants of the OmpF protein which itself anchors onto the gold substrate via coordination of the cysteine thiol group [85].

## 4. Conclusions

Australia and New Zealand, in particular, are home to some of the world’s leading researchers into the use of tethered bilayer lipid membranes for antimicrobial research. Australia is also the home of the world’s only commercial supplier of tethered bilayer lipid membranes. In this work, we have reviewed how this technology has been used to assist in identifying how antimicrobial agents interact with lipid bilayers and, where appropriate, highlighted the works of the southern hemisphere research groups who are the leaders in this field of research.
